# Influence of Gypsum Type on Early Hydration Kinetics and Autogenous Shrinkage of Supersulfated Cement-Based UHPC Matrix

**DOI:** 10.3390/ma19101985

**Published:** 2026-05-11

**Authors:** Yuanwei Ju, Anming She, Junyan Wang

**Affiliations:** Key Laboratory of Advanced Civil Engineering Materials of Ministry of Education, School of Materials Science and Engineering, Tongji University, Shanghai 201804, China; 2331543@tongji.edu.cn (Y.J.)

**Keywords:** supersulfated cement, ultra-high-performance concrete, hydration, autogenous shrinkage, gypsum type

## Abstract

Ultra-high-performance concrete (UHPC) matrix faces critical challenges of high carbon footprint and significant autogenous shrinkage. Supersulfated cement (SSC), a potentially lower-carbon binder comprising ground granulated blast-furnace slag and gypsum, offers a promising alternative. This study systematically investigated the effect of gypsum type—phosphogypsum (PG), dihydrate gypsum (DH), and anhydrite (AH)—on the early hydration and shrinkage behavior of UHPC matrix incorporating 30% SSC as Portland cement replacement. A multi-technique approach, including mechanical testing, isothermal calorimetry, XRD, TG-DSC, SEM, LF-NMR, and autogenous shrinkage measurements, was employed. Results demonstrate that gypsum type critically governs sulfate dissolution kinetics, thereby dictating phase assemblage and microstructural evolution. DH provides relatively rapid sulfate dissolution, promoting earlier AFt and gel formation, which is associated with the highest early strengths and a marked reduction in autogenous shrinkage. AH shows a slower but sustained sulfate supply, resulting in comparable 28-day strength with moderate shrinkage reduction. PG yielded the lowest autogenous shrinkage (374 μm/m at 7 d), but it also suffered from severe early-age retardation due to soluble phosphate impurities, as evidenced by the delayed hydration peak and lowest 3 d strength. This behavior is mainly related to strong early-age retardation, delayed hydration, delayed setting, and a prolonged low-stiffness state. These findings suggest that appropriate gypsum selection in SSC enables tailored early-age performance and improved volume stability in the UHPC matrix, offering guidance for utilizing industrial by-products such as phosphogypsum in sustainable high-performance concrete design.

## 1. Introduction

Ultra-high-performance concrete (UHPC) is widely recognized as an advanced cementitious material, characterized by exceptional mechanical properties (with compressive strength typically exceeding 120 MPa) [1,2,3,4], superior durability, and a dense microstructure [2]. However, compared with normal concrete (NC), conventional UHPC formulations employ significantly higher amounts of binder (800–1100 kg/m^3^), approximately three to four times that of NC [5], resulting in a correspondingly higher carbon footprint during material design [5,6,7], which contradicts the global pursuit of sustainability in the construction sector. Additionally, the combination of high cement content and low water-to-binder ratio in UHPC leads to pronounced autogenous shrinkage [8,9,10]. Previous studies have reported that UHPC exhibits autogenous shrinkage in the range of 500–800 μm/m within the first 24 h after the onset of hydration, increasing the risk of cracking and jeopardizing the long-term stability and durability of structures [11,12,13,14]. Therefore, to mitigate both the environmental impact and autogenous shrinkage of UHPC, researchers have focused on incorporating low-carbon substitutes [7], utilizing industrial by-products or supplementary cementitious materials (SCMs), such as fly ash (FA) or ground granulated blast-furnace slag (GGBS) [15]. Consequently, the development of UHPC mixtures that simultaneously exhibit a low carbon footprint and reduced shrinkage has become a central focus in civil engineering materials research.

Supersulfated cement (SSC) consists of 70–90% GGBS, 0–20% sulfate activators (typically gypsum), and a small amount of alkali activators [16,17,18,19]. SSC has been reported to show substantially lower CO_2_ emissions than ordinary Portland cement (OPC), in some studies by about 80–88% [20,21]. SSC-based materials are characterized by low hydration heat, good chemical resistance, and long-term strength development, making them an attractive option for sustainable concrete applications. Recent studies have explored the incorporation of SSC into UHPC, confirming its potential to reduce the carbon footprint without significantly compromising performance, while substantially mitigating early-age autogenous shrinkage. Within SSC systems, gypsum serves as the primary sulfate source and plays a critical role in activating slag by promoting the formation of ettringite (AFt) and C–S–H gel. The hydration products are rich in AFt, which induces micro-expansion, thereby alleviating shrinkage issues commonly observed in conventional cement systems. The specific reactions are summarized as follows [22]:

(1) $${Ca\left(OH\right)}_{2}+{SiO}_{2}\to C-S-H\left(gel\right)$$

(2) $${Ca\left(OH\right)}_{2}+{Al}_{2}O_{3}\to C-A-H\left(gel\right)$$

(3) $$C-A-H\left(gel\right)+{CaSO}_{4}+H_{2}O\to AFt$$

(4) $${Ca\left(OH\right)}_{2}+{Al}_{2}O_{3}+3{CaSO}_{4}+H_{2}O\to AFt$$

The type and source of gypsum have a significant impact on its dissolution rate, sulfate release efficiency, and subsequent hydration kinetics. Common gypsum variants include dihydrate gypsum (CaSO_4_·2H_2_O), anhydrite (CaSO_4_), and industrial by-products such as phosphogypsum (primarily CaSO_4_·2H_2_O with trace impurities). Phosphogypsum, a major industrial waste from phosphoric acid production, offers potential environmental benefits if effectively utilized; however, its impurities (e.g., phosphates, fluorides) may alter hydration pathways [23,24,25]. It is noteworthy that phosphogypsum (PG), as an industrial by-product, is classified as technologically enhanced naturally occurring radioactive material (TENORM), and its radioactivity mainly originates from the uranium and thorium decay series present in the phosphate rock. In this study, the phosphogypsum was subjected to a washing pretreatment, and the resulting material complies with the Chinese national standard building gypsum (GB/T 9776-2022 [26]), which specifies the limits for radionuclides in gypsum-based construction materials, thereby ensuring its radiological safety for building applications. Previous studies have demonstrated that, through appropriate pretreatment processes (e.g., washing, neutralization) and dosage control, phosphogypsum incorporated into cement-based materials can effectively immobilize hazardous elements and maintain radionuclide levels within acceptable limits, meeting radiological safety requirements for construction materials [27]. Studies have shown that SSC containing phosphogypsum generates greater amounts of C–S–H and AFt at later hydration stages, leading to a denser microstructure [28]. Anhydrite can delay hydration, whereas dihydrate gypsum accelerates early hydration. The content of sulfate activator influences both the formation and stability of AFt [29]. Furthermore, when the calcium sulfate content is low (~10%), the lack of SO_4_^2−^ leads to the conversion of AFt into AFm [30]. Conversely, higher calcium sulfate contents result in increased AFt precipitation on the surface of slag particles. Due to the expansive effect of AFt, cracks may form in the hydrated SSC paste, reducing the strength of SSC [31].

Despite these advances, systematic comparisons of the role of gypsum type in SSC-based UHPC matrices remain relatively limited, particularly under ultra-low water-to-binder ratio conditions. Most existing studies have focused on SSC in conventional concrete or mortar systems with higher water-to-binder ratios, where hydration conditions differ markedly from the ultra-low water content and high packing density characteristic of the UHPC matrix. As a result, the influence of different gypsum sources—such as phosphogypsum (PG), dihydrate gypsum (DH), and anhydrite (AH)—on sulfate release behavior, early-age phase assemblage, and microstructural development in such dense systems has not been consistently evaluated under comparable conditions. In particular, while the retarding effect of impurities in phosphogypsum is generally recognized, its combined influence on hydration timing, early mechanical development, and autogenous shrinkage behavior in SSC-UHPC systems remains insufficiently documented in a systematic manner. To address these gaps, this study aims to investigate how different types of gypsum affect the early hydration kinetics and phase composition evolution of low water-to-binder ratio ultra-high-performance concrete (UHPC) matrices, and how the timing of AFt formation relative to matrix setting influences autogenous shrinkage. To explore these issues, the present study adopts an established UHPC matrix mix design and replaces 30% of white Portland cement with SSC, systematically varying only the gypsum type (PG, DH, AH) while maintaining a control group without additional gypsum (SSC-0). Fiber-free mixtures are investigated to isolate binder effects and reduce complexity. A multi-technique approach—including mechanical testing, isothermal calorimetry, XRD, TG-DSC, SEM, low-field NMR, and autogenous shrinkage measurements—is employed to evaluate hydration kinetics, phase development, microstructure, pore structure evolution, and macroscopic performance.

## 2. Experimental Program

### 2.1. Materials

The materials used in this study are as follows: white Portland cement (WPC, CEM I 52.5, density 3036 kg/m^3^, Jiangxi Yinshan White Cement Co., Ltd., Jinggangshan, Jiangxi, China); ground granulated blast-furnace slag (GGBS, grade S105, density 2990 kg/m^3^, specific surface area > 500 m^2^/kg, Shanghai Siqi Building Materials Co., Ltd., Shanghai, China); limestone powder (LP, density 2699 kg/m^3^, Henan Panlong New Materials Co., Ltd., Nanyang, Henan, China); pretreated phosphogypsum (PG, density 2656 kg/m^3^, pretreat phosphogypsum by washing with water, Changde Hongguan Gypsum Environmental Protection Materials Technology Co., Ltd., Changde, Hunan, China); dihydrate gypsum (DH, density 2320 kg/m^3^, Xilong Scientific Co., Ltd., Shantou, Guangdong, China); anhydrite (AH, density 2960 kg/m^3^, Xilong Scientific Co., Ltd., Shantou, Guangdong, China); and fly ash cenospheres (FAC, density 2520 kg/m^3^). Both DH and AH were of analytical grade with a purity exceeding 99.0%. The chemical compositions of the other raw materials are summarized in Table 1. The chemical composition of the raw material was determined by X-ray fluorescence (XRF) analysis. Fine aggregates were quartz sand sieved to 20–40 mesh and 40–80 mesh fractions (Shanghai Botong Chemical Co., Ltd., Shanghai, China). A powdered polycarboxylate-based high-range water reducer (SP) was used as the superplasticizer, and a solid organic-silicone-based defoamer was employed as the air-entrainment control agent. All mixing was performed using ordinary tap water.

**Figure 1:**
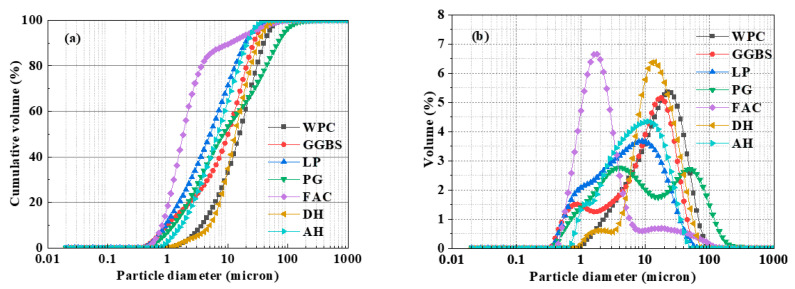
Particle size distribution of raw materials: (**a**) cumulative distribution curve of raw material particle size; (**b**) particle size distribution curve of raw materials.

**Figure 2:**
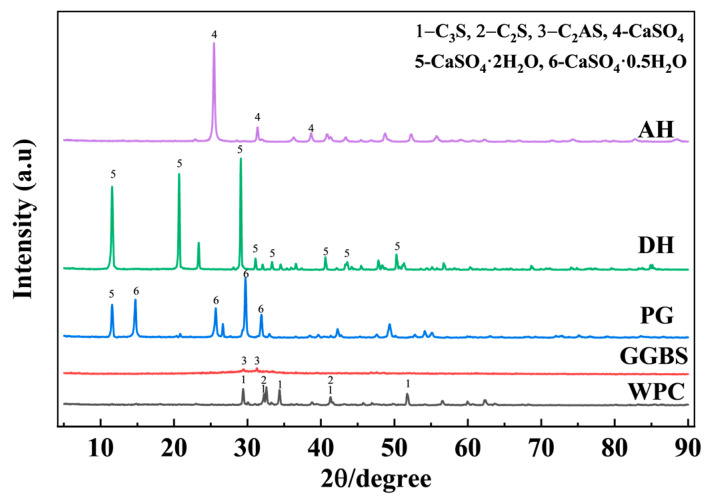
XRD pattern of the pristine raw materials.

The following symbols are defined: WPC represents the ordinary white Portland cement; GGBS denotes the content of ground granulated blast-furnace slag; PG refers to the dosage of pretreated phosphogypsum; LP indicates the amount of limestone powder; FAC represents the content of fly ash cenospheres; SSC denotes the content of supersulfated cement, which is itself a mixture of WPC, PG (DH or AH), and GGBS in a mass ratio of 5%, 20%, and 75%, while the overall binder system also contains additional GGBS, limestone powder, and fly ash cenospheres. SSC-PG indicates that the gypsum type in SSC is PG. The particle size distribution of the raw materials is shown in Figure 1. The XRD patterns of the raw materials are presented in Figure 2.

### 2.2. Specimen Preparation

Based on preliminary tests, the mix proportions were designed as summarized in Table 2. Three types of gypsum in SSC (phosphogypsum, dihydrate gypsum, and anhydrite) were investigated to evaluate their effects on UHPC performance. In all mixtures, the total mass of the two cementitious components was normalized to 1.0, and the masses of all other components were expressed as a ratio relative to this baseline. Representative mix designs are presented in Table 2. The water-to-binder ratio was fixed at 0.22, while the total volume of binder, batch water content (kg/m^3^), and sand content (kg/m^3^) were kept constant. The control mixture, denoted SSC-0, contains no SSC replacement. In the other mixtures, 30% of the white Portland cement was replaced by SSC, with only the gypsum type varying. For example, SSC-PG indicates that 30% of the mass of white Portland cement in the binder system is replaced by SSC, in which the gypsum type is phosphogypsum.

The mixing procedure was as follows: pre-weighed powders were added to the mixer and dry-mixed at low speed for 1 min; water was then added and mixed at low speed for 3 min; quartz sand was subsequently incorporated and mixed at low speed for 4 min; finally, the mixture was mixed at high speed for 4 min. Fresh specimens were cast into molds, covered with plastic film, and left undisturbed for 24 h before demolding. All specimens were then cured under standard conditions (20 ± 1 °C, relative humidity ≥ 98%) until the designated testing ages.

### 2.3. Methods

#### 2.3.1. Mechanical Performance Testing

According to the national standard GB/T 17671-2021 [32], prismatic specimens (40 mm × 40 mm × 160 mm) and cubic specimens (40 mm × 40 mm × 40 mm) were prepared. At each designated curing age, compressive and flexural strengths were measured using a fully automatic 100-ton constant-stress testing machine. To ensure statistical reliability, six identical cubic specimens were tested for compressive strength at each age, and the reported values are the arithmetic average of the six measurements. Flexural strength was determined using three identical prismatic specimens per age, and the mean of three measurements was reported.

#### 2.3.2. Isothermal Calorimetry

All powders and deionized water were equilibrated at 20 ± 1 °C for 24 h prior to testing. For isothermal calorimetry, 10 g of total powder (WPC, GGBS, PG/DH/AH, LP, FAC, and SP) was weighed for each mixture, dry-mixed on weighing paper, and transferred to ampoule vials. The corresponding mass of deionized water was added with a pipette, and the mixture was manually stirred with a rod for 60 s. The ampoules were placed in an eight-channel TAM AIR isothermal calorimeter (TA Instruments, New Castle, DE, USA), and heat flow data were recorded every 30 s for 72 h.

#### 2.3.3. X-Ray Diffraction (XRD) Analysis

X-ray diffraction (XRD) measurements were performed using a Rigaku D/max-2200PC diffractometer (Rigaku Corporation, Tokyo, Japan) to characterize hydration kinetics and products. The instrument was operated with Cu radiation (λ = 1.54056 Å) at 35 kV and 25 mA. XRD patterns were collected over a 2θ range from 5° to 90° with a step size of 0.02° and a scanning rate of 5°/min. The obtained diffraction patterns were used to identify the crystalline phases present in the samples. Hardened paste samples were collected at 3 and 7 days, and hydration was stopped by immersion in anhydrous ethanol. The samples were then dried in an oven at 40 °C for 24 h, ground in an agate mortar for 30 min, and sieved through a 200-mesh screen.

#### 2.3.4. Thermogravimetric–Differential Scanning Calorimetry (TG–DSC)

Thermogravimetric and differential scanning calorimetry (TG–DSC) tests were conducted on a TA Instruments SDT Q600 (TA Instruments) under a nitrogen atmosphere. Samples were heated from room temperature to 1000 °C at a rate of 10 °C/min. Following [33,34], chemically bound water (*W_CBW_*) and calcium hydroxide content (*W_CH_*) were calculated from the TG curves, where *W*_30–500 °C_ represents the mass loss (%) in the 30–500 °C range of the cement mortar TG curve, and W_400–500 °C_ represents the mass loss (%) in the 400–500 °C range:

(5) $$W_{CBW}=W_{30-500\;{}^{\circ}C}$$

(6) $$W_{CH}=\frac{74}{18}\times W_{400-500\;{}^{\circ}C}$$

Here, *W*_30–500 °C_ represents the mass loss (%) of cement mortar specimens in the 30–500 °C range of the TG curve, and *W*_400–500 °C_ represents the mass loss (%) in the 400–500 °C range.

#### 2.3.5. Scanning Electron Microscopy (SEM)

To investigate the effect of SSC on early hydration products, fracture surfaces with flat, representative morphology were selected from specimens at 3 days of age. Samples were gold-coated to enhance conductivity and imaged using a Zeiss Sigma 300 scanning electron microscope (Carl Zeiss Microscopy GmbH, Jena, Germany) to visualize morphology and microstructural features.

#### 2.3.6. Low-Field Nuclear Magnetic Resonance (LF-NMR)

Early hydration kinetics and pore size evolution were probed using low-field nuclear magnetic resonance (LF-NMR) with a 12 MHz NMR spectrometer (NMRC 12-010-T, Niumag, Suzhou, China) under a 0.28 ± 0.05 T magnetic field and a 10 mm coil. Transverse relaxation (*T*_2_) of ^1^H nuclei was measured using the Carr–Purcell–Meiboom–Gill (CPMG) pulse sequence [35,36]. The relaxation time of water molecules in the cement paste reflects their state [36]. LF-NMR measurements primarily rely on the transformation of physically bound water in the paste, providing insight into the phase evolution of the cementitious system. Freshly mixed paste was transferred into sample vials, sealed, and placed in the dedicated measurement well. Data were collected at 10 min intervals over at least 72 h. *T*_2_ distribution spectra were obtained from the decay curves using the simultaneous iterative reconstruction technique (SIRT) [37,38].

#### 2.3.7. Setup for Shrinkage Testing

Autogenous shrinkage tests were conducted following GB/T 50082 [39], using an HC-NES non-contact sensor system [40]. This system allows synchronous recording of free deformation, internal specimen temperature, ambient temperature, and relative humidity. Prismatic molds measured 100 mm × 100 mm × 515 mm. Specimens for autogenous shrinkage measurements were embedded with a vinyl film to minimize moisture evaporation. For each mixture, three independent specimens were measured, and the reported shrinkage curves represent the average of these three replicates. All specimens were continuously cured under standard conditions (20 °C, 98% relative humidity) for 7 days to monitor shrinkage behavior.

#### 2.3.8. Initial Setting Time Testing

This study was conducted in accordance with ASTM C403/C403M standard [41], using a penetration resistance apparatus (Hebei Yiqishun Testing Instrument Co., Ltd., Cangzhou, Hebei Province, China) to monitor the setting evolution process of UHPC paste. Given that the UHPC mixture contains no coarse aggregate, the fresh mortar, after excluding steel fibers, was directly placed into standard molds. The tests were carried out under constant temperature and humidity conditions (20 ± 2 °C, RH > 50%). By using needles with different cross-sectional areas, the resistance values were recorded periodically when the penetration depth reached 25 mm. The initial setting time was defined as the time point corresponding to a penetration resistance of 3.5 MPa, which was determined by nonlinear regression analysis of the experimental resistance–time data.

## 3. Results and Discussion

### 3.1. Mechanical Properties

**Figure 3:**
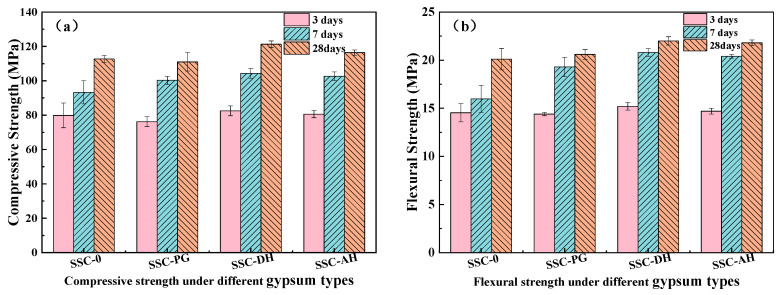
Mechanical properties of UHPC matrix with different gypsum types: (**a**) compressive strength; (**b**) flexural strength.

The type of gypsum significantly influences the temporal evolution of compressive and flexural strengths in SSC-based UHPC matrix. The underlying mechanisms primarily stem from differences in gypsum dissolution kinetics, sulfate availability, and the timing of hydrate formation. Figure 3a shows the compressive strength development of SSC-UHPC specimens made from different types of gypsum at 3 days, 7 days, and 28 days. At an early age (3 days), the compressive strength of SSC-DH (82.5 MPa) was slightly higher than that of SSC-0 (79.8 MPa) by 2.7 MPa, whereas the compressive strength of SSC-PG was lower than that of the reference group. This is mainly attributed to dihydrate gypsum (DH) possessing the most suitable dissolution rate, which rapidly releases ${SO}_{4}^{2-}$ ions, promoting the swift formation of an AFt framework that fills the matrix pores [29,42]. In contrast, SSC-PG exhibited the lowest 3-day strength, primarily due to the retarding influence of soluble phosphates and fluorides, as evidenced by the prolonged induction period observed in isothermal calorimetry. At 7 days and 28 days, the SSC-incorporated groups generally showed comparable or higher compressive strength development compared to SSC-0, with SSC-DH reaching 104.3 MPa (7 d) and 121.3 MPa (28 d). Although SSC-PG recovered considerably by 28 days, its compressive strength remained slightly lower than SSC-0, possibly due to residual effects of impurities (e.g., phosphate-related phases) that may have introduced some microstructural heterogeneity.

The flexural strength results (Figure 3b) reveal the following trends: at early ages, SSC-DH exhibited the highest flexural strength, reaching 20.8 MPa at 7 days, which was 5.8 MPa higher than that of the reference group. Moreover, all groups incorporating SSC showed higher flexural strengths than the reference group. This trend suggests that the incorporation of SSC may promote the formation of a microstructure characterized by the intergrowth of acicular AFt crystals and C-S-H gel, as observed under scanning electron microscopy (SEM). Compared to the CH-dominated microstructure in SSC-0, this structural configuration likely contributes to improved crack-bridging capacity and enhanced flexural performance. Furthermore, the moderate expansion behavior observed in SSC-DH and SSC-AH may partially compensate for the tensile stresses induced by autogenous shrinkage, which could help explain the better retention of flexural strength in these mixtures. In summary, the use of DH and AH types of SSC appears to enhance both the early-age and long-term mechanical properties of the UHPC matrix in a balanced manner, potentially due to the timely formation of AFt, densification of the microstructure, and a certain degree of shrinkage compensation.

### 3.2. Heat of Hydration

**Figure 4:**
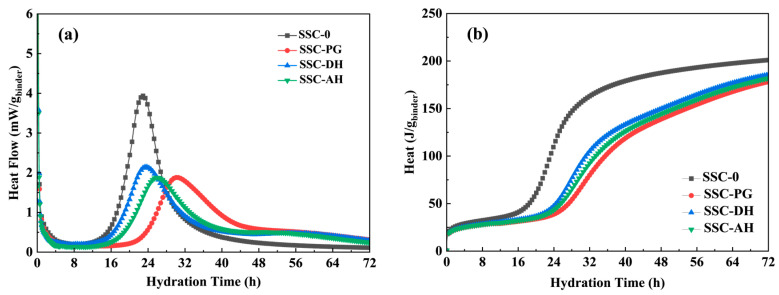
Hydration heat of cementitious materials for UHPC matrix under different gypsum types: (**a**) heat flow; (**b**) cumulative heat.

Figure 4a,b illustrates the heat flow and cumulative heat release of the UHPC pastes within the first 72 h. All mixtures exhibited a typical exothermic process at early ages: an initial peak appeared within minutes after mixing, associated with particle wetting, dissolution, and rapid aluminate reactions in the presence of sulfates; subsequently, a main peak occurred, corresponding to the acceleration period of silicate hydration and significant C-S-H formation [43]. The timing of the main hydration peak varied depending on the gypsum type. SSC-0, SSC-DH, SSC-AH, and SSC-PG reached their second exothermic peaks at 23.2 h, 23.5 h, 26.1 h, and 30.1 h, respectively. In addition to this dominant peak, a weak late sulfate/aluminate-related feature was observed at around 56 h. This feature was most clearly resolved in SSC-AH and was also present in SSC-DH, although it was less distinguishable because of its lower intensity and partial overlap with the deceleration-stage heat flow in the combined plot. The clearer separation in SSC-AH is consistent with the slower dissolution kinetics of anhydrite, which temporally decoupled the sulfate-related aluminate reaction from the main acceleration peak. By contrast, in SSC-DH, the faster sulfate release caused a larger fraction of sulfate–aluminate reactions to occur earlier and overlap with the dominant peak. In SSC-PG, the corresponding signal appeared only as a very weak hump, indicating that the sulfate-related reaction was substantially weakened and broadened. In SSC-0, where no additional gypsum was introduced, no clearly separated late peak could be resolved, suggesting that the corresponding sulfate–aluminate reaction was either weaker or embedded within the initial and main exothermic events. The nearly simultaneous peaks of SSC-DH and SSC-0 indicate that dihydrate gypsum can provide sulfate ions relatively quickly, allowing the system to enter the acceleration phase rapidly, even with a 30% SSC blend. The moderate delay of the main peak in SSC-AH is consistent with the slower dissolution kinetics of anhydrite, leading to a sustained but delayed release of sulfate ions. The significant delay observed in SSC-PG (30.1 h, approximately 6.9 h later than SSC-0) aligns with the known effects of soluble impurities (phosphates) in phosphogypsum, which prolong the induction period by reducing the alkalinity of the pore solution. The inhibition by PG is not merely a kinetic delay, but a thermodynamic suppression. It is known that soluble phosphates from PG can complex with Ca^2+^ ions and adsorb on the surface of clinker and slag [44], effectively increasing the activation energy of nucleation at phase boundaries, resulting in a significant delay and broadening of the hydration peak, and a notable prolongation of the initial setting time (Table 3). The delayed hydration in SSC-PG corresponds to a lower degree of early-age microstructure development and the lowest 3-day strength, whereas the timely peak in SSC-DH suggests earlier formation of hydration products.

Differences were observed in the cumulative heat release within 72 h: SSC-0 released 201.1 J/g, SSC-PG released 178.3 J/g, while the cumulative heat releases of SSC-DH and SSC-AH were also generally consistent. This phenomenon can be explained by (i) the 30% SSC blend reduces the proportion of high-heat clinker phases, consistent with the typically lower hydration heat of supersulfated systems, and (ii) strength depends more on the type, formation timing, and spatial distribution of hydration products than on total heat release. Accordingly, the timely sulfate supply in the SSC-DH and SSC-AH groups is considered to favor earlier sulfate–aluminate reactions and earlier development of C-(A)-S-H-rich binding phases, as indirectly indicated by calorimetry and corroborated by the subsequent XRD, SEM, and NMR results, despite the relatively lower cumulative heat. SSC-PG exhibited an early delay affecting its 3-day strength, but subsequently recovered by 28 days, consistent with a delayed but ongoing hydration process. Subsequent XRD, SEM, and NMR analyses were broadly consistent with this interpretation that the DH and AH groups exhibited more timely AFt and gel formation and a finer pore structure, whereas SSC-PG showed relatively delayed early product formation and a coarser pore structure during the initial stages.

### 3.3. X-Ray Diffraction (XRD)

**Figure 5:**
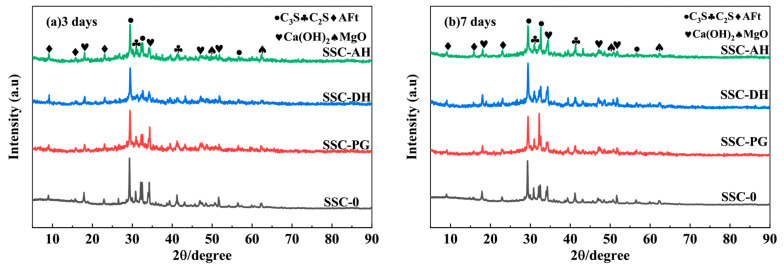
XRD of cementitious materials for UHPC matrix under different gypsum types: (**a**) 3 days, (**b**) 7 days.

It should be noted that, in the absence of Rietveld refinement, the XRD results in this study are used only for qualitative phase identification and discussion of relative phase-evolution trends, rather than for quantitative phase fraction determination. The mineralogical compositions of the raw materials, including PG, DH, AH, cement, and GGBS, were characterized by XRD and are presented in Figure 2, providing a basis for interpreting their reactivity and subsequent hydration behavior. The XRD patterns of the UHPC pastes at 3 days and 7 days (Figure 5) reveal generally similar mineral assemblages, while differences in the reflections of Portlandite (CH) and AFt qualitatively suggest that the hydration process was affected by the gypsum type. At 3 days, the control group (SSC-0) exhibited the strongest CH peaks, consistent with the substantial Portlandite generation from the hydration of predominantly C_3_S and C_2_S phases. In contrast, all groups incorporating SSC (SSC-DH, SSC-AH, and SSC-PG) showed lower CH peak intensities than SSC-0. This trend may be qualitatively associated with the dilution of clinker phases and the consumption of CH through subsequent reactions involving slag and fly ash; however, no quantitative phase content is inferred from peak intensity alone. This observation is also qualitatively consistent with the scanning electron microscopy (SEM) images, which show that the large CH crystals (~10 μm) in SSC-0 were replaced by a denser matrix in the SSC groups. Notably, among the SSC groups, SSC-DH exhibited the strongest AFt reflections at 3 days, qualitatively suggesting more pronounced early AFt formation and crystallization. This is consistent with the rapid sulfate supply provided by dihydrate gypsum, which supports the formation of early hydration products and contributes to early compressive and flexural strength [45]. In contrast, SSC-PG displayed weaker AFt peaks compared to SSC-DH at day 3, likely reflecting the inhibitory effect of soluble phosphates in phosphogypsum on the nucleation and growth of hydrates.

By day 7, the AFt reflections became more evident in all SSC groups, while the CH peaks remained relatively suppressed, indicating the continuation of sulfate-activated reactions. The transition from a pattern dominated by CH peaks in the SSC-0 group to a system with enhanced AFt peaks in the SSC-DH and SSC-AH groups suggests continued sulfate-activated hydrate formation and a greater contribution of AFt-containing hydrates. These XRD observations should be interpreted qualitatively and in conjunction with the calorimetry, SEM, and NMR results, rather than as a standalone quantitative assessment of phase assemblage.

### 3.4. TG–DSC

**Figure 6:**
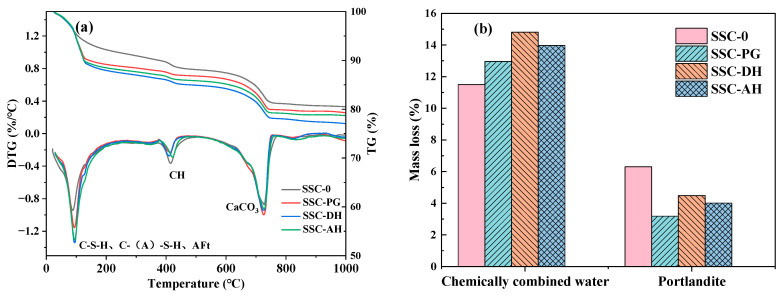
TG/DTG results of sample at (**a**) 3 d DTG-TG and (**b**) sample chemically bound water and calcium hydroxide content.

As shown in the TG-DTG curves (Figure 6a,b), all 3 d samples exhibit several distinct mass loss peaks. The three most prominent peaks correspond to [46] (1) the main peak in the 100–150 °C region, which arises from overlapping dehydration processes of C-S-H, C-(A)-S-H, AFt, and AFm phases, rather than a single phase transition; (2) a peak in the 400–500 °C region, associated with dehydroxylation of Ca(OH)_2_ (CH); and (3) a peak in the 600–750 °C region, arising from decomposition of CaCO_3_, which likely forms through sample carbonation.

It should be noted that, within the 100–200 °C range, multiple dehydration events overlap. In particular, a shoulder feature can be observed at approximately ~130 °C in the DTG curves, which is commonly reported in the literature as indicative of the presence of AFm phases or the overlapping dehydration of AFt and AFm [46]. Typically, AFt dehydrates at lower temperatures (~80–120 °C), whereas AFm phases dehydrate at slightly higher temperatures (~120–180 °C), leading to the formation of composite peaks with shoulder characteristics [43]. Therefore, the main peaks in this region, as shown in Figure 6, are mainly attributed to the dehydration of AFt and C-(A)-S-H, while the shoulder peak at about 130 °C is temporarily related to the dehydration process associated with AFm. Analysis of the thermogravimetric data at 3 days indicates that the hydration kinetics were strongly influenced by the dissolution and release characteristics of the sulfate component. The order of chemically bound water (*W_CBW_*) content directly reflects the degree of hydration and the generation of water-rich phases. It should be noted that part of this measured chemically bound water in SSC-DH and SSC-PG may originate from the inherent hydration of CaSO_4_·2H_2_O present in the raw materials. However, the trends observed by TG remain consistent with the apparent hydration degree measured by LF-NMR, and the comparative interpretation among different gypsum types is not affected. The SSC-DH group exhibited the highest *W*_CBW_ value (14.81%), which was higher than that of the control group SSC-0 (11.5%), confirming that the rapid dissolution of dihydrate gypsum, combined with aluminate reactions, promoted the extensive formation of water-rich hydrates such as AFt and C-S-H gel. The lower *W_CBW_* value of the SSC-AH group reflects a kinetic delay caused by the slow dissolution of anhydrite, which limited the local sulfate concentration required for AFt nucleation.

The control group (SSC-0) exhibited the lowest *W_CBW_* value, attributable to the stoichiometric differences in hydration products: the Portland cement matrix primarily generates C-S-H and CH, whose water-binding capacity is significantly lower than that of the AFt-dominated phases in the SSC system. This is consistent with the lowest apparent hydration measured by LF-NMR. The extremely high CH content in SSC-0 confirms that, in the absence of slag activation, CH merely accumulates as a by-product of tricalcium silicate (C_3_S) hydration without being consumed by significant pozzolanic reaction. Notably, the CH content of SSC-DH was higher than that of SSC-AH, suggesting that in the DH system, the accelerating effect of rapid sulfate supply on C_3_S hydration temporarily generated CH at a rate exceeding its consumption by the pozzolanic reaction of the slag. In contrast, the lower CH content in SSC-AH may not stem from efficient pozzolanic consumption, but rather from the delayed clinker hydration caused by slow anhydrite dissolution, which reduced the initial generation of CH. The SSC-PG group exhibited both low CH content and low *W_CBW_*, consistent with the PG-induced hydration delay shown in Figure 6b. The soluble phosphates in phosphogypsum adsorb onto cement particle surfaces, forming a metastable passivation layer that inhibits C_3_S dissolution. Consequently, the low CH content in SSC-PG does not result from depletion by pozzolanic components, but rather reflects a state of chemical inhibition with a prolonged induction period.

These thermogravimetric analysis results corroborate the isothermal calorimetry findings: DH facilitates the timely formation of water-rich hydrates with a balanced generation and consumption of CH, AH exhibits a delayed but progressive hydration process, while PG is subject to early chemical inhibition, with its low CH content being a manifestation of the extended induction period.

### 3.5. SEM Results

**Figure 7:**
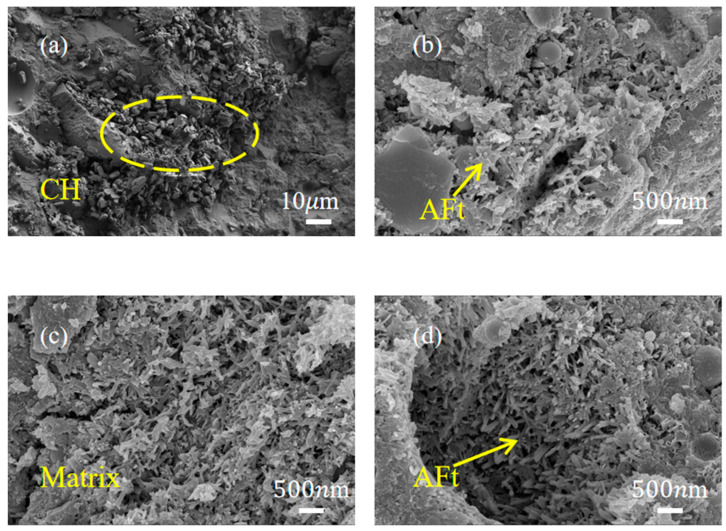
SEM images of 3 d age specimens (**a**) SSC-0; (**b**) SSC-PG; (**c**) SSC-DH; (**d**) SSC-AH.

SEM microstructural observations at different scales reveal differences in microstructural features among the UHPC mixtures that are consistent with variations in mechanical performance and hydration kinetics. At a 10 µm scale (Figure 7a), the control group (SSC-0) shows a microstructure dominated by large, hexagonal portlandite (CH) crystals within a relatively porous matrix. The presence of these coarse CH crystals aligns with the relatively high cumulative heat release observed in calorimetry, reflecting predominant C_3_S hydration in Portland cement that produces significant CH as a by-product rather than extensive gel phases. These plate-like CH crystals often appear at interfaces and in the matrix, which may contribute to weaker zones in the interfacial transition zone (ITZ), potentially explaining the lower flexural strength observed in SSC-0 despite its higher early heat evolution. In contrast, the SSC-incorporated groups (SSC-DH, SSC-AH, and SSC-PG) observed at higher magnification (500 nm scale) exhibit a microstructure characterized by abundant needle-like AFt crystals intergrown with C-S-H gel (Figure 7b–d). In particular, SSC-DH displays a dense microstructure with extensive needle-like AFt crystals that appear closely associated with the surrounding C-S-H gel, which likely contributes to the higher early compressive and flexural strengths observed in this group. The rapid sulfate supply from dihydrate gypsum in SSC-DH appears to support timely slag activation and AFt formation while limiting excessive CH accumulation. Although SSC-AH and SSC-PG also show AFt formation, the degree of integration between AFt crystals and the gel matrix appears less pronounced in these groups compared to SSC-DH, consistent with their relatively delayed or inhibited early hydration kinetics. These microstructural differences support the overall trends in mechanical performance and hydration behavior, indicating that appropriate gypsum selection influences the timing and morphology of key hydration products, contributing to balanced strength development and volume stability.

### 3.6. LF-NMR

The NMR-derived hydration degree (HD) can be estimated for relative comparison by using the intensity of the relaxation signal from LF NMR results [47,48]:

(7) $$\alpha_{t}=\frac{I_{0}-I_{t}}{I_{0}}*\frac{X}{\gamma }$$

where $I_{0}$ represents the water signal intensity at the initial hydration stage, $I_{t}$ denotes the signal intensity at time t, γ is the water-to-cement ratio required for complete cement hydration (0.38 data is mainly based on approximate calculations from the literature, and is mainly used here for relative comparisons between mixtures rather than as an absolute hydration degree, given the complexity and multi-component nature of SSC-UHPC) and  X  is the experimental w/c ratio.

In low-field NMR measurements using a standard CPMG sequence, both chemically bound water and a significant portion of gel water become invisible due to their extremely short transverse relaxation times (*T*_2_). Therefore, the theoretical water requirement for complete hydration (γ) must account for both components. Based on the revisited Powers–Brownyard model [48,49], the γ value for ordinary Portland cement is taken as 0.42. For supplementary cementitious materials, the water-binding capacity is fundamentally different. According to the stoichiometric frameworks proposed by Merzouki et al. [50] and Chen and Brouwers [51], the γ value for blast furnace slag was determined to be approximately 0.32. Similarly, based on the investigations by Lam et al. [52] and Wang and Lee [53], the γ value for fly ash was taken as 0.20. Inert fillers, such as limestone powder, consume negligible water during hydration, and their γ values were set to 0. The overall γ for the UHPC mixture was calculated using a mass-weighted average of the individual binder components.

(8) γ=∑piγi

In this study, *γi* represents the water demand of individual components: for Portland cement, *γ_WPC_* = 0.42 (based on the Powers model); for slag, *γ_GGBS_* ≈ 0.32; for fly ash, *γ_FAC_* ≈ 0.20; and limestone powder is assumed to be inert, with *γ_LP_* ≈ 0.

**Figure 8:**
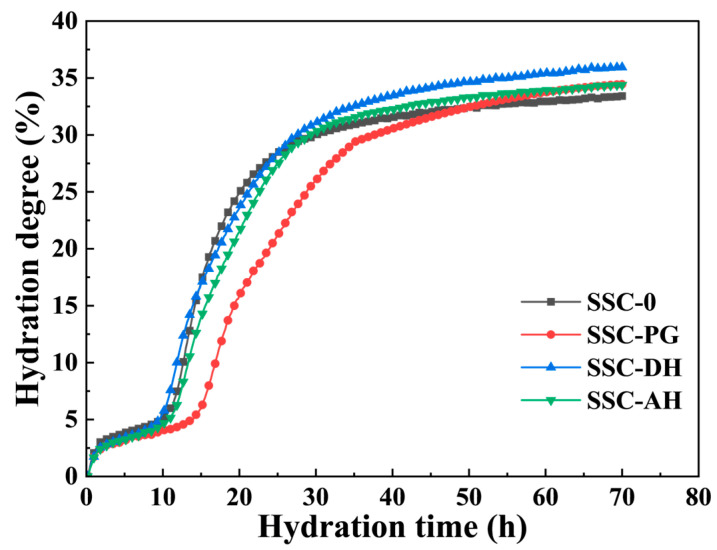
The hydration degree of each sample group over time.

**Figure 9:**
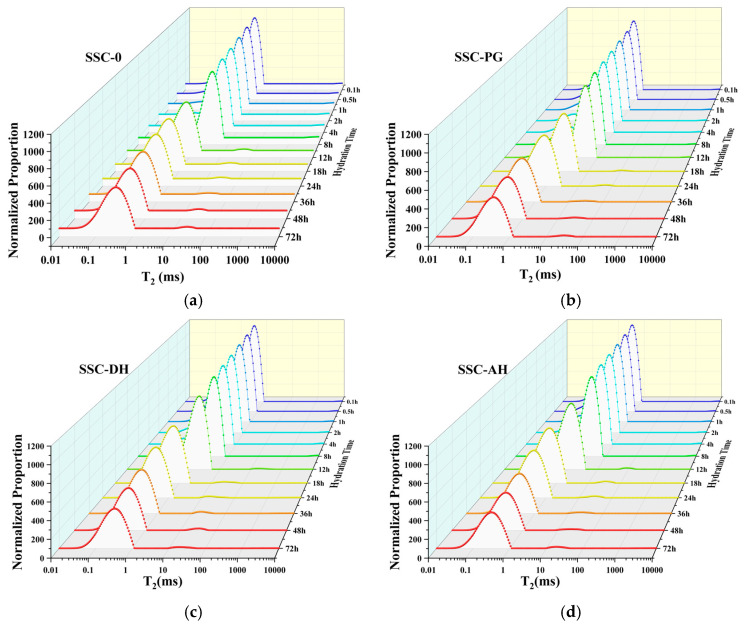
*T*_2_ spectra of each sample group at specific times within 72 h; (**a**) SSC-0; (**b**) SSC-PG; (**c**) SSC-DH; (**d**) SSC-AH.

Figure 8 presents the evolution of UHPC hydration degree over time based on the evaporable water EW relaxation signals in cement pastes. At 72 h, SSC-DH exhibits the highest *α_t_* value, although remaining below 50% due to ongoing hydration and continuous water consumption in the low w/b system. While SSC incorporation may influence initial hydration rates—with SSC-0 showing relatively higher *α_t_* than SSC-AH and SSC-PG during some early periods—the calculated *α_t_* values at 3 d increase with SSC addition. Specifically, SSC-DH reaches an *α_t_* of 35.9%, approximately 2.6 percentage points higher than the control (SSC-0). This trend is consistent with the higher chemically bound water content observed in SSC-containing specimens by TG analysis. This difference can be attributed to the substantial formation of AFt in SSC systems. AFt incorporates a large amount of water (32 molecules per mole), which contributes significantly to the measured bound water. Thus, even if silicate phase hydration proceeds more slowly in early stages due to SSC effects, the abundant AFt formation can lead to higher apparent *α_t_* values overall, as reflected in the NMR-derived index. These results align with the complementary calorimetry, XRD, and TG findings, indicating that gypsum type influences the timing and type of hydration products, with DH supporting relatively higher early bound water incorporation through timely AFt and C-S-H formation. Figure 9 displays the *T*_2_ distribution spectra of each sample group at selected time points from initial mixing to 72 h. Within the first approximately 2 h, all groups exhibit a single peak in the 1–10 ms range. As hydration proceeds, partially physically bound water transforms into chemically bound water, manifesting as peak attenuation accompanied by leftward peak migration—gradually shifting toward 1 ms, with the majority of peaks distributed in the 0.03–1 ms range and a minor fraction remaining in the 1–10 ms range.

According to LF-NMR theory [54], physically bound water can be categorized into three types based on binding degree: free water, water in capillary pores, and water in gel pores. As hydration progresses, free water rapidly converts to water in capillary pores (also termed interlayer water) and subsequently transforms into water in gel pores (also termed nanopore water) [55], driving the observed leftward peak shift. Comparison of *T*_2_ spectra between SSC-0 and other sample groups reveals that the SSC-DH specimen exhibits a progressive leftward shift in the *T*_2_ peak at 72 h, indicating an increasing proportion of gel pores in SSC-DH. This demonstrates that DH promotes hydration development, with hydration products in SSC-DH generating finer pore structures within the specimen interior. This finding correlates well with the enhanced early compressive strength, confirming that SSC incorporation at early ages elevates hydration degree through the high water demand of its hydration products while preserving the UHPC pore structure—with SSC-DH exhibiting the most pronounced effect.

This rapid evolution of water states and pore refinement during the first 72 h is closely related to the development of autogenous shrinkage. The shrinkage measurements indicate that approximately 79–87% of the total 7-day autogenous shrinkage occurs within the first 3 days, which coincides with the period of most pronounced water redistribution observed by LF-NMR. The conversion of free water into capillary and gel-bound water reduces internal relative humidity and enhances capillary stresses, thereby acting as a primary driving force for early-age autogenous shrinkage. LF-NMR results provide a mechanistic link between hydration kinetics, pore structure evolution, and autogenous shrinkage behavior, highlighting that early-age water redistribution plays a dominant role in controlling shrinkage development in SSC-based UHPC systems.

### 3.7. Autogenous Shrinkage Measurement

**Figure 10:**
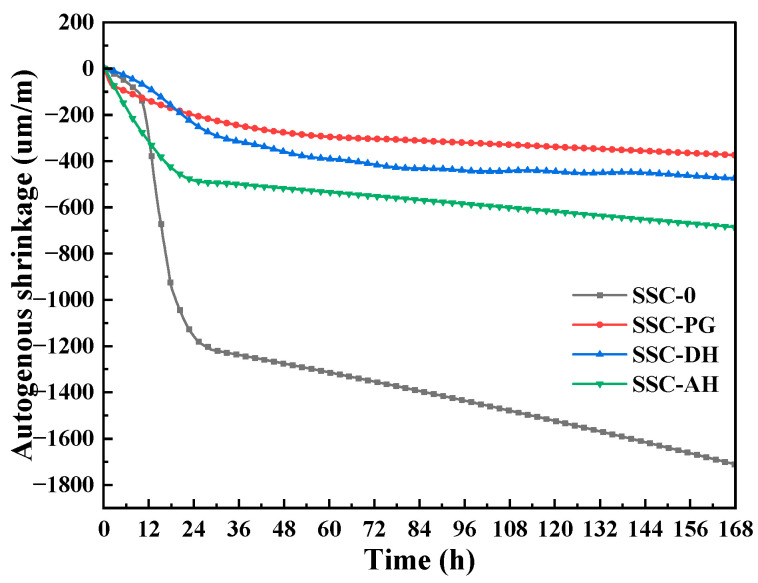
Autogenous shrinkage strain versus age of UHPC matrix with different gypsum types.

Autogenous shrinkage was measured from the initial setting time, with downward strain indicating shrinkage and upward strain indicating expansion. The initial setting times for the four groups are shown in Table 3. Figure 10 shows the 7-day autogenous shrinkage characteristics of the SSC-based UHPC matrix. This volume contraction arises from rapid hydration of the high Portland cement content, leading to self-desiccation in the fine pore network and development of capillary tension in the absence of compensatory mechanisms. Incorporation of SSC reduces autogenous shrinkage compared to the control, with the extent and underlying mechanisms varying by gypsum type. SSC-DH (474.7 μm/m) and SSC-AH (685.1 μm/m) show reduced shrinkage likely associated with expansion from AFt formation. In SSC-DH, rapid sulfate release from dihydrate gypsum supports relatively timely AFt crystallization during the hardening period, which may contribute to counteracting tensile stresses from self-desiccation. This results in a shrinkage reduction of 72.2% relative to SSC-0, while maintaining relatively high early strength. The moderately higher shrinkage in SSC-AH is consistent with slower anhydrite dissolution, leading to delayed AFt formation relative to matrix stiffening, potentially reducing the effectiveness of early expansion. SSC-PG exhibits the lowest measured shrinkage (374 μm/m), although this appears primarily attributable to significant early hydration retardation. While the XRD analysis indicates the presence of hemihydrate in PG, which typically accelerates setting due to its high solubility, the overall hydration kinetics of the SSC-PG system are dominated by the inhibitory effects of soluble phosphates (0.67% $P_{2}O_{5}$ as shown in Table 1). These impurities significantly delay the initial setting and the overall hydration progression [23,44], as evidenced by the delayed secondary heat peak and lower early strength. The prolonged low-stiffness state allows chemical shrinkage to be accommodated through bulk deformation rather than inducing significant autogenous strain.

Thus, unlike the expansion-related mitigation observed in SSC-DH and SSC-AH, the low shrinkage in SSC-PG likely reflects delayed structural development rather than active volume compensation, which is consistent with its relatively poor early-age mechanical performance. These results indicate that gypsum type influences autogenous shrinkage through differences in sulfate release kinetics, AFt formation timing, and overall hydration progression, offering options for balancing early strength and volume stability in SSC-based UHPC matrix.

## 4. Conclusions

This study examined the influence of gypsum type—phosphogypsum (PG), dihydrate gypsum (DH), and anhydrite (AH)—on the early hydration behavior, microstructural evolution, and macroscopic properties of supersulfated cement-based UHPC matrix. The conclusions are limited to the investigated formulation and conditions. The main conclusions are as follows:

(1) Gypsum type influences the early hydration kinetics of SSC-based UHPC matrix by affecting sulfate dissolution rates. DH shows relatively rapid dissolution, releasing SO_4_^2−^ ions to support slag hydration, with the second exothermic peak at 23.5 h and relatively high early bound water content at 3 d. AH exhibits slower dissolution, with a delayed second peak at 26.1 h, consistent with sustained but gradual sulfate release. PG, due to the presence of soluble phosphate and fluoride impurities, prolongs the induction period, shifting the second peak to 30.1 h and resulting in lower early hydration extent.
(2) Hydration product assemblage and microstructural development are strongly influenced by gypsum type. XRD and SEM reveal that SSC-0 is dominated by large CH crystals (~10 μm), while SSC-DH develops abundant, well-crystallized AFt intergrown with C-S-H gel at 3 d. SSC-AH shows similar features but with delayed formation. SSC-PG exhibits limited early AFt, consistent with its retarded hydration. TG analysis quantifies these differences: SSC-DH achieves the highest chemically bound water at 3 d (14.81%), confirming abundant water-rich hydrate formation, while SSC-0 shows the lowest (11.50%), suggesting more pronounced early hydration in the DH system.
(3) LF-NMR provides supportive evidence regarding hydration-related water consumption and pore-environment evolution that underpin the macroscopic performance. At 72 h, SSC-DH exhibits the highest hydration degree, consistent with its high early strength and chemically bound water. *T*_2_ relaxation spectra show that SSC-DH develops the finest pore structure, with peaks shifting toward shorter relaxation times, indicating an increased proportion of gel pores. SSC-AH shows intermediate pore refinement, while SSC-PG retains a coarser pore structure at early ages, reflecting its delayed hydration.
(4) SSC incorporation reduces autogenous shrinkage of the UHPC matrix by 58.2–78.1% at 7 d compared to the control. The mechanisms vary with gypsum type. SSC-DH and SSC-AH show reduced shrinkage that may be associated with AFt formation during hardening, which may contribute to compensating some self-desiccation stresses, with DH exhibiting greater reduction (72.2% at 7 d) due to more timely AFt development. PG achieves passive shrinkage reduction (78.1% at 7d), but this behavior is primarily attributed to strong early-age retardation rather than an intrinsic shrinkage-compensation effect, as delayed setting prolongs the plastic phase and reduces post-set hydration rate, leading to compromised early strength.

In summary, the selection of gypsum type in SSC allows regulation of early hydration kinetics, microstructural features, and volume stability in the UHPC matrix. These findings provide guidance for developing UHPC mixtures with improved strength development and shrinkage control while incorporating industrial by-product gypsum. Further work covering a wider range of SSC contents and mixture designs is needed to generalize these findings.

## Figures and Tables

**Table 1:** Chemical composition of raw materials (wt %).

Component	CaO	SiO_2_	Al_2_O_3_	Fe_2_O_3_	MgO	Na_2_O	K_2_O	SO_3_	P_2_O_5_
WPC	64.51	21.32	4.61	3.20	1.32	0.14	0.66	2.21	-
GGBS	41.04	31.57	14.06	0.65	8.52	0.48	0.28	2.13	-
PG	43.99	6.05	0.25	0.06	0.03	-	0.10	48.44	0.67
LP	50.29	1.37	0.43	0.15	4.40	-	0.08	-	-
FAC	9.13	57.53	18.20	4.96	2.12	2.18	2.54	0.24	-

**Table 2:** Mix design example of UHPC mortar specimens.

Materials	SSC-0	SSC-PG	SSC-DH	SSC-AH
WPC	1	0.7	0.7	0.7
SSC:PG	0	0.06	0	0
SSC:DH	0	0	0.06	0
SSC:AH	0	0	0	0.06
SSC:GGBS	0	0.225	0.225	0.225
SSC:WPC	0	0.015	0.015	0.015
GGBS	0.369	0.369	0.369	0.369
LP	0.166	0.166	0.166	0.166
FAC	0.049	0.049	0.049	0.049
20–40 Meshes Sand	0.881	0.881	0.881	0.881
40–80 Meshes Sand	0.881	0.881	0.881	0.881
Water	0.295	0.295	0.295	0.295
SP	0.010	0.010	0.010	0.010
Defoamer	0.001	0.001	0.001	0.001

**Table 3:** The initial setting times for different samples.

Groups	SSC-0	SSC-PG	SSC-DH	SSC-AH
Times (h)	8.7	10.3	11.7	13.6

## Data Availability

The original contributions presented in this study are included in the article. Further inquiries can be directed to the corresponding author.
